# Effects of human and organizational deficiencies on workers’ safety behavior at a mining site in Iran

**DOI:** 10.4178/epih.e2018019

**Published:** 2018-05-18

**Authors:** Mostafa Mirzaei Aliabadi, Hamed Aghaei, Omid Kalatpour, Ali Reza Soltanian, Maryam SeyedTabib

**Affiliations:** 1 Center of Excellence for Occupational Health Engineering, Occupational Health and Safety Research Center, School of Public Health, Hamadan University of Medical Sciences, Hamadan, Iran; 2 Center of Excellence for Occupational Health Engineering, School of Public Health and Research Center for Health Sciences, Hamadan University of Medical Sciences, Hamadan, Iran; 3 Department of Biostatistics and Epidemiology, School of Public Health and Modeling of Non-Communicable Diseases Research Center, Hamadan University of Medical Sciences, Hamadan, Iran

**Keywords:** Mining accident analysis, Human factors, Behavior, Statistical model

## Abstract

**OBJECTIVES:**

Throughout the world, mines are dangerous workplaces with high accident rates. According to the Statistical Center of Iran, the number of occupational accidents in Iranian mines has increased in recent years. This study investigated and analyzed the human and organizational deficiencies that influenced Iranian mining accidents.

**METHODS:**

In this study, the data associated with 305 mining accidents were analyzed using a systems analysis approach to identify critical deficiencies in organizational influences, unsafe supervision, preconditions for unsafe acts, and workers’ unsafe acts. Partial least square structural equation modeling (PLS-SEM) was utilized to model the interactions among these deficiencies.

**RESULTS:**

Organizational deficiencies had a direct positive effect on workers’ violations (path coefficient, 0.16) and workers’ errors (path coefficient, 0.23). The effect of unsafe supervision on workers’ violations and workers’ errors was also significant, with path coefficients of 0.14 and 0.20, respectively. Likewise, preconditions for unsafe acts had a significant effect on both workers’ violations (path coefficient, 0.16) and workers’ errors (path coefficient, 0.21). Moreover, organizational deficiencies had an indirect positive effect on workers’ unsafe acts, mediated by unsafe supervision and preconditions for unsafe acts. Among the variables examined in the current study, organizational influences had the strongest impact on workers’ unsafe acts.

**CONCLUSIONS:**

Organizational deficiencies were found to be the main cause of accidents in the mining sector, as they affected all other aspects of system safety. In order to prevent occupational accidents, organizational deficiencies should be modified first.

## INTRODUCTION

The mining industry plays a crucial role in the economy of countries throughout the world. Despite extensive efforts to improve safety in mines, accidents remain a threat to the sustainability of the mining industry, as they can lead to injuries and death among workers, property degradation, and damage to the environment [1]. According to previous studies, the rate of accidents in the mining industry is very considerable [2].

Several studies have investigated the main causes of accidents in the mining industry. A study of mining accidents in the US attributed nearly 85% of mine accidents to human error [1]. Since human behavior in a complex socio-technical system such as the mining industry is affected by various organizational and environmental factors, it cannot be correct to consider human error to be the main cause of accidents [3]. Some studies have reported that inappropriate working conditions, such as rock falls and explosions, were the main causes of mining accidents [4,5]. A number of studies have identified inadequacies in management measures, such as a lack of proper training courses for workers and inadequate supervision, as the main causes of mining accidents [4,6,7]. However, a comprehensive analysis for identifying the causal factors of accidents has not been conducted yet. Determining the interactions between causal factors of accidents can lead to better comprehension of the defects in the system. System-based accident analysis models can help to understand why accidents occur, and identifying patterns of accidents would be helpful for preventing their reoccurrence [2].

Multiple system-based accident analysis models have been developed [8,9]. The human factors analysis and classification system (HFACS) is one of the most widely used and reliable accident analysis models [10], and was developed by Wiegmann & Shappell [3] based on the Swiss cheese model [9]. Instead of introducing humans as the main cause of accidents, the HFACS systematically seeks to recognize the active and latent errors in the system that can eventually result in an accident. The HFACS model categorizes 19 causal factors in a hierarchical order of 4 levels of human and organizational failures, including unsafe acts, preconditions for unsafe acts, unsafe supervision, and organizational influences. In this approach, investigators first identify the types of unsafe acts that are thought to lead to the accident, and then look for the root causes of the accident at the organizational and supervisory levels. Based on the HFACS approach, at least 1 deficiency is present at each level when an accident occurs. If the organization corrects any of the failures that results in an accident, the accident will be avoided.

The HFACS was first introduced to analyze aviation accidents [3], and then has been employed in other areas such as marine accidents [11,12], clinical errors [13,14], and rail incidents [15,16]. Some studies have utilized the HFACS for analyzing mining accidents. Patterson & Shappell [17] applied a modified version of the HFACS to analyze 508 mining accidents with the goal of recognizing the human and organizational deficiencies that led to mining accidents in Queensland. In another study, Lenné et al. [18] used the original HFACS to characterize the relationships of organizational and supervisory failures with unsafe acts of the operator in mining accidents. Verma & Chaudhari [19] analyzed human factors contributing to Indian manganese mining accidents using a modified version of the HFACS. However, no studies have conducted a comprehensive analysis to determine the relationships and interactions among the human and organizational deficiencies that contribute to mining accidents.

According to the Statistical Center of Iran, Iran had 5,316 active mines in 2012, from which more than 60 minerals were extracted. Iran is among the top 15 mineral-rich countries, and more than 100,000 people are employed in the mining industry directly and approximately 2 million more people indirectly. Unfortunately, the number of occupational accidents in Iranian mines has increased from 876 cases in 2009 to 1,177 cases in 2012 [20,21]; however, a large number of work-related accidents may has not been reported by organizations. Thus, the present study focused on analyzing Iranian mining accidents based on the HFACS model to identify human and organizational deficiencies and to determine the interactions between these deficiencies by using partial least square structural equation modeling (PLS-SEM).

Structural equation modeling (SEM) is a powerful analysis tool from the multivariable regression family that allows a set of regression equations to be tested simultaneously. SEM is a comprehensive statistical approach for testing hypotheses about relationships between observed and latent variables [22,23]. Covariancebased structural equation modeling (CB-SEM) and PLS-SEM are the 2 main approaches for estimating SEM [24]. In the CB-SEM approach, the proposed model is examined using a covariance matrix, while in the PLS-SEM approach, the model is assessed by describing the variance in the variables. PLS-SEM is recommended when the data do not follow a multivariate normal distribution, few data are available, and the model is formative [25].

## MATERIALS AND METHODS

### Data source and coding process

Reports of 305 significant mining accidents involving considerable property damage, fatality, or serious injuries occurring between 2002 and 2016 and results of the accident analysis were gathered from one of the largest iron ore mines in Kerman, Iran. The company employed an accident analysis team with various experts to analyze significant accidents. Accident analysis was performed using the root cause analysis (RCA) approach. RCA is a problem-solving approach utilized to identify the root causes of problems [26].

As mentioned earlier, the HFACS has 4 levels. Level 1 is workers’ unsafe acts and includes 2 categories: workers’ errors and workers’ violations. An error is an unintentional action of a worker that can result in an accident, while a violation is an intentional disregard of rules and regulations that can also result in an accident [3]. Violation should not be conflated with sabotage, because the former category includes actions that generally have the goal of completing tasks faster and in a more effective manner, while the latter category refers to actions carried out with the aim of damaging the system. Level 2 contains the preconditions for unsafe acts, including various physical and technological factors, adverse mental/physiological states, and physical limitations of workers that can contribute to an unsafe act or accident. Preconditions for unsafe acts include environmental factors, condition of operator, and personnel factors [3]. Level 3 corresponds to unsafe supervision, which refers to supervisors’ disregard of safety problems in the workplace. Unsafe supervision is subdivided into 4 subcategories: inadequate supervision, planned inappropriate operation, failed to correct known problem, and supervisory violations [3]. Level 4 refers to organizational influences, or deficiencies at the top management level that affect the likelihood of an accident. Organizational influences are subdivided into 3 subcategories: resource management, organizational climate, and operational process [3].

In the present study, workers’ unsafe acts, preconditions for unsafe acts, unsafe supervision, and organizational influences were regarded as latent variables, and categories related to each variable that could be determined directly were regarded as their indicators. The latent variables and their related indicators are presented in Table 1.

Each indicator was regarded as a binary variable, for which a score of 0 meant that the indicator did not contribute to the accident, and a score of 1 meant that the indicator contributed to the accident. To determine the score of each indicator in each accident (the coding process), 2 ergonomists with 4 years of experience in the mining industry were asked to assign a score to the variables based on accident data, such as accident report forms, photographs of the accident, and RCA results. All accidents were coded based on the above-described procedure and a database was created. The coding process was performed by consensus to increase inter-rater reliability.

### Building the model

SEM models are normally constructed based on the hypotheses that are to be examined. Since a previous study [1] demonstrated that 85% of mining accidents occurred due to workers’ unsafe acts, in the present study, the influence of system deficiencies on workers’ unsafe acts was evaluated based on the following hypotheses:

Hypothesis 1: Organizational influences exert a direct impact on unsafe acts of workers; Hypothesis 2: The effect of organizational influences on unsafe acts of workers is mediated by unsafe supervision; Hypothesis 3: The effect of organizational influences on unsafe acts of workers is mediated by preconditions for unsafe acts; Hypothesis 4: Organizational influences have an impact on unsafe acts of workers through a sequence of unsafe supervision and preconditions for unsafe acts.

**Figure 1.**
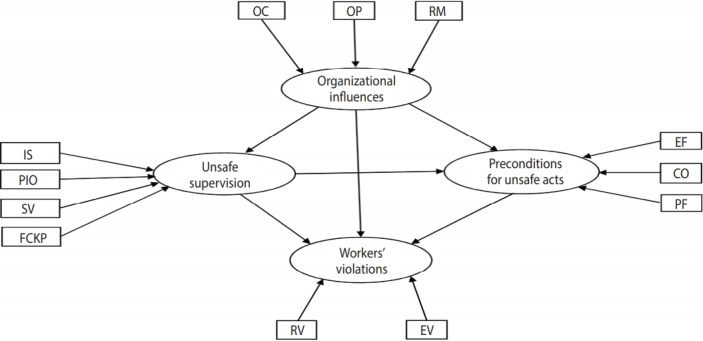
The hypothesis model for describing the influences of casual factors on workers’ violations. OC, organizational climate; OP, organizational process; RM, resource management; IS, inadequate supervision; PIO, planned inappropriate operation; SV, supervisory violation; FCKP, failed to correct known problem; EF, environmental factors; CO, condition of operator; PF, personnel factors; RV, routine violations; EV, exceptional violations.

**Figure 2.**
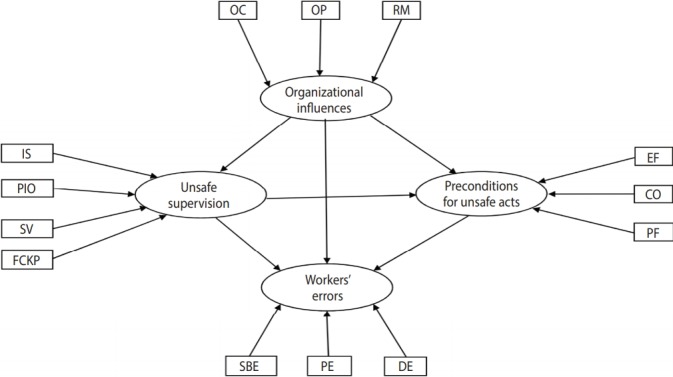
The hypothesis model for describing the influences of casual factors on workers’ errors. OC, organizational climate; OP, organizational process; RM, resource management; IS, inadequate supervision; PIO, planned inappropriate operation; SV, supervisory violation; FCKP, failed to correct known problem; EF, environmental factors; CO, condition of operator; PF, personnel factors; SBE, skill-based errors; DE, decision errors; PE, perceptual errors.

Since workers’ unsafe acts can be divided into errors and violations, we examined the above hypotheses for workers’ violations and for workers’ errors separately. The proposed models are illustrated in Figures 1 and 2 for workers’ violations and workers’ errors, respectively.

The database was imported into SmartPLS 2.0 (https://www.smartpls.com/smartpls2) and the hypotheses of the present study were examined using the bootstrap method. Since the applied model in this study was formative and few data were available, PLSSEM was employed [25]. Following Zhang et al. [27], the process of data analysis with PLS-SEM for verifying the theoretical model was performed in 2 steps. In the first step, the quality of the measurement model (individual indicator validity) was evaluated. Various indices were used to assess the measurement model according to the type of the indicators in the model [28]. Since the proposed models in the present study had formative indicators, we used the indicator weight to determine which indicators should be eliminated and which should be retained in the model [28]. Accordingly, after running PLS-SEM, the significant indicators and the non-significant ones weighted more than 0.5 were retained in the model, while the others were excluded.

**Figure 3.**
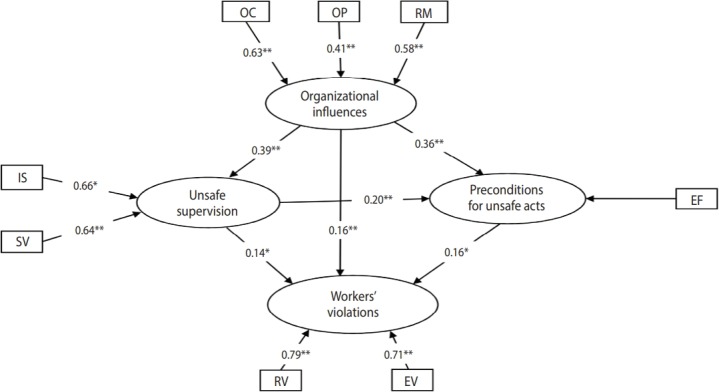
Final PLS-SEM model of workers’ violations with indicator weights and path coefficients among latent variables. PLS-SEM, partial least square structural equation modeling; OC, organizational climate; OP, organizational process; RM, resource management; IS, inadequate supervision; SV, supervisory violation; EF, environmental factors; RV, routine violations; EV, exceptional violations. *p<0.05, **p<0.01.

**Figure 4.**
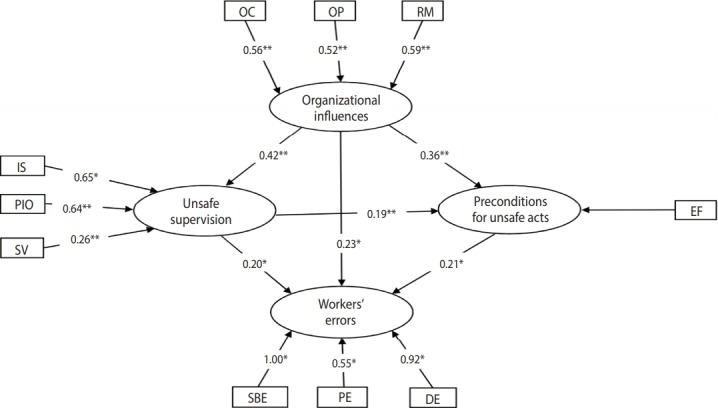
Final PLS-SEM model of workers’ errors with indicator weights and path coefficients among latent variables. PLS-SEM, partial least square structural equation modeling; OC, organizational climate; OP, organizational process; RM, resource management; IS, inadequate supervision; SV, supervisory violation; PIO, planned inappropriate operation; EF, environmental factors; SBE, skill-based errors; PE, perceptual errors; DE, decision errors. *p<0.05, **p<0.01.

After determining the indicators that needed to be retained in the 2 models, the data were analyzed again, and new indicator weights and t-values were calculated (Figures 3 and 4). As shown in Figures 3 and 4, all indicators in the 2 models were significant (p< 0.05). After evaluating the quality of the measurement model, the second step was to evaluate the structural model (construct validity). In this step, using R^2^ , cross-validated redundancy (CV-Red), cross-validated communality (CV-Com), and goodness-of-fit (GoF) indices that were fitted to the model with formative indicators [25,28], the quality of the structural model was evaluated. R^2^ is a measure of the variance explained in each of the endogenous latent variables. CV-Com assesses how much each latent variable is useful to the model adjustment and CV-Red assesses the accuracy of the adjusted model. GoF assesses the quality of the adjusted model [25]. Table 2 show the results of structural evaluation of the models of workers’ violations and workers’ errors, respectively.

As Table 2 shows, CV-Red and CV-Com were acceptable for all latent variables in the model of workers’ violations. Additionally, R^2^ values were 15.48, 23.25, and 13.50%, respectively, for unsafe supervision, preconditions for unsafe acts, and workers’ violations. Table 2 shows that CV-Red and CV-Com were acceptable for all latent variables in the model of workers’ errors. Unsafe supervision (R^2^ , 18.10%), preconditions for unsafe acts (R^2^ , 22.32%), and workers’ errors (R^2^ , 13.22%) had a medium effect on workers’ errors. We determined the GoF for the workers’ violations and workers’ errors models to be 0.32 and 0.28, respectively, which indicated that the 2 models had an adequate adjustment [29].

The study protocol was approved by the Hamadan University of Medical Sciences (ethical code: IR.UMSHA.REC.1395.458).

## RESULTS

A total of 305 mining accidents were investigated. All the workers involved in the accidents were men, with a mean age of 33.0±7.6 years and an average experience of 7.6± 4.3 years. Moreover, 76% of them were married and the rest were single.

Before interpreting the results of PLS-SEM, the quality of the proposed models was assessed in 2 steps and the suitability of the proposed models was confirmed (Figures 3 and 4). Based on Figure 3, among the 3 indicators of the organizational influences variable, the organizational climate had the greatest effect (indicator weight, 0.63) and the organizational process had the least effect (indicator weight, 0.41). Inadequate supervision (indicator weight, 0.66) and supervisory violations (indicator weight, 0.64) had a nearly equal influence on the unsafe supervision variable. Since among all the indicators of the preconditions for unsafe acts variable, only environmental factors were retained in the workers’ violations model, this indicator had the greatest effect on preconditions for unsafe acts. Of the 2 indicators of the workers’ violations variable, routine violations had a greater effect than exceptional violations.

In the workers’ errors model (Figure 4), resource management had the most influence on the organizational influences, while organizational process had the least. Planned inappropriate operation and inadequate supervision had a large effect on unsafe supervision, whereas supervisory violations had a small effect. Furthermore, environmental factors were the indicator with the greatest effect on the preconditions for unsafe acts. Among the 3 indicators of the workers’ errors variable, skill-based errors had the greatest effect and perceptual errors had the least effect.

Hypotheses 1 to 4 aimed at assessing the interactions among unsafe acts of workers and organizational influences, unsafe supervision, and preconditions for unsafe acts. As shown in Figure 3, workers’ violations were positively affected by the organizational influences (path coefficient, 0.16) and this effect was statistically significant (p< 0.01). Therefore, hypothesis 1 was confirmed for the workers’ violations model. Moreover, the impact of organizational influences on workers’ violations was mediated by unsafe supervision because the path from organizational influences to unsafe supervision and the path from unsafe supervision to workers’ violations were statistically significant; hence, hypothesis 2 was supported. Additionally, as shown in Figure 3, the impact of organizational influences on workers’ violations was mediated by preconditions for unsafe acts (environmental factors), thereby confirming hypothesis 3. Furthermore, organizational influences impacted workers’ violations through a sequence of unsafe supervision and preconditions for unsafe acts; therefore, hypothesis 4 was confirmed (Figure 3).

Figure 4 shows the interactions among latent variables in the model of workers’ errors. Organizational influences had a direct positive effect (path coefficient, 0.23) on workers’ errors, and this effect was statistically significant (p< 0.05), confirming hypothesis 1. Organizational influences had a significant effect on unsafe supervision (p< 0.01) and unsafe supervision had a statistically significant effect on workers’ errors (p< 0.05), thereby supporting hypothesis 2. Hypothesis 3 was confirmed, because the impact of organizational influences on preconditions for unsafe acts was statistically significant, as was the impact on preconditions for unsafe acts on workers’ errors. Finally, organizational influences impacted errors through the sequence of unsafe supervision and preconditions for unsafe acts to a statistically significant extent; therefore, hypothesis 4 was also supported.

## DISCUSSION

Workers’ unsafe acts are usually considered to be the main cause of industrial accidents. However, in complex systems, humans are only one of multiple mutually-interacting system components. In the analysis of unsafe acts, these interactions should be taken into account. Therefore, several studies have suggested that accidents should be investigated and analyzed using systematic methods to recognize deficiencies in all components of the system [32,33]. A comprehensive investigation of accidents using systematic analysis methods can help to gain a deep understanding of the relationships and interactions among system components [34]. In the present study, mining accidents were analyzed using the HFACS as a reliable system-based method, and interactions between human and organizational factors were examined with PLS-SEM.

In the present study, based on the PLS-SEM results, hypotheses 1 to 4 for the models of workers’ unsafe acts were supported. According to hypothesis 1, organizational deficiencies have a direct effect on workers’ unsafe acts. Some studies [27,35] demonstrated that organizational factors such as deficiencies in safety management, organizational involvement, and unsafe rules directly affected workers’ unsafe acts. Moreover, other studies [36,37] showed that safety culture and work pressure were organizational factors that affected workers’ unsafe acts. Time pressure and a high workload can affect human error and the efficiency of operators [38].

Hypothesis 2 states that organizational deficiencies exert an effect on workers’ unsafe acts mediated by unsafe supervision. According to the psychosocial model of workplace accidents, the organizational safety climate influences workers’ safety behavior, mediated by supervisors’ safety responses [39]. Therefore, the role of supervisors as a mediator between management rules/policies and workers can have a crucial effect on workers’ safe acts. Indeed, supervisors can provide a supportive environment for safety in which safety behavior is encouraged. Several studies have explained that one of the main reasons why workers do not engage in safe behavior is the fear of being teased by coworkers [40]. Another reason is that some workers regard safety behavior as a sign of weakness [41]. Both these examples are indicative of a workplace with a poor supportive environment. However, it should be noted that the lack of a supportive environment is a sign of poor management commitment to safety. In other words, when safety is important for managers, it is important for supervisors too, thus resulting in a supportive environment for safety behavior [42]. Indeed, management commitment to safety is crucial for providing a supportive environment for safety. Moreover, when supervisors ignore safety rules and regulations, frontline employees may lose their motivation to engage in safe behavior; in such a situation, reward and punishment systems are also meaningless and the effectiveness of incentive systems cannot be maintained in the long term [43].

In this study, hypothesis 3 was also confirmed, meaning that managerial lack of attention towards solving environmental and technological problems, such as lack of funding for the necessary technological tools for the workplace, can lead to workers’ unsafe acts. For this reason, mines are harsh and polluted workplaces. In such a poor working environment, workers are uncomfortable and tend to take shortcuts when performing their tasks, consequently ignoring safety issues and increasing the risk of accidents. This pattern has also been observed among outdoor workers. Several studies have found the rate of unsafe behavior to increase during the middle of the day when the outdoor temperature is at its peak [44]. Likewise, the finding is in accordance with the results obtained by Ramsey et al. [45], who found that the rate of safety behavior decreased as the thermal conditions of the workplace deviated from the optimum range. In the same vein, the effect of environmental factors on safety behavior has also been investigated by some other studies [46]. Therefore, more attention from management to housekeeping, ventilation systems, and thermal comfort may be helpful as a way to decrease accidents by improving environmental factors.

Finally, hypothesis 4 was supported, indicating that organizational influences affect unsafe acts of workers through a sequence of unsafe supervision and preconditions for unsafe acts. In fact, organizational factors such as management commitment to safety are essential for improving health and safety issues in workplaces. Without management commitment, safety programs are unlikely to succeed [47]. Inadequate staffing is another organization-related factor that can affect safety behavior and accidents [48]. When the number of workers in the workplace is lower than what is needed, workers must work fast, perform several tasks simultaneously, and take shortcuts. Consequently, their attention decreases and the probability of error increases. Moreover, this result is in agreement with the study by Lenné et al. [18] that analyzed major mining accidents based on the HFACS model and found that there was a significant relationship among certain contributing factors in the organization, supervision, preconditions for unsafe acts, and workers’ unsafe act levels. System-based accident analysis models suggest that the elimination of latent errors across a sophisticated system is the most appropriate strategy for preventing accidents, instead of addressing the active errors of workers. This is also in accordance with the conceptual model proposed by Neal et al. [49], which argues that organizational climate is the root cause of poor safety performance.

Some strengths and limitations of this study should be acknowledged. In the present study, many mining accidents were investigated, meaning that the sample size was a major strong point of the study. Another strong point was the use of a systematic and well-accepted framework as a basis for the PLS-SEM model. However, this study also had some limitations that should be mentioned. A major limitation is that the accident data investigated in the present study were obtained from a single mining site in Iran; hence, it is recommended for future studies to consider more accidents from various mining sites. Moreover, although PLS-SEM is a powerful method for interaction analysis, other methods, such as Bayesian networks [50], are recommended for developing predictive models.

In conclusion, organizational deficiencies were found to be the main causes of mining accidents. These deficiencies have both direct and indirect effects on unsafe acts. Organizational deficiencies can also lead to unsafe supervision and preconditions for unsafe acts. Without modifying these deficiencies, attempts to prevent mining accidents would probably fail to achieve the desired results. Therefore, in order to reduce the rate of mining accidents, deficiencies at higher organizational levels should be addressed.

## Figures and Tables

**Table 1.** Latent variables of study and their related indicators

Latent variable	Indicator	Description
Workers’ errors	Skill-based errors	Errors that occur in highly routine operations, due to workers’ attention deviation
	Decision errors	This error type occurs when the action of a worker is intentional, while the provided plan to achieve the desired outcome is inadequate
	Perceptual errors	Errors that occur when the sensory input of a worker is degraded and an action is taken based on defective information
Workers’ violations	Routine violations	Habitual behavior of a worker that is tolerated by the management
	Exceptional violations	Violations committed by a worker in abnormal situations
Preconditions for unsafe acts	Environmental factors	A variety of issues involving conditions of the physical environment (e.g., heat and lighting) and technological environment (e.g., design of equipment)
	Condition of operator	Includes issues such as adverse mental states, adverse physiological states, and physical/mental limitations of workers
	Personnel factors	Refers to lack of communication and teamwork among individuals
Unsafe supervision	Inadequate supervision	Refers to situations in which the provided supervision is not effective
	Planned inappropriate operation	Refers to operational tempo or work scheduling that puts workers at risk or affects their performance
	Failed to correct known problem	Occurs when defects related to the safety domain such as equipment and staff are known to supervisors, yet are permitted to continue
	Supervisory violations	The intentional disregard of supervisors for existing rules and instructions
Organizational influences	Resource management	The human, monetary, and equipment resources that management allocates to safety issues
	Organizational climate	Refers to the atmosphere within the organization such as culture, policies, and structure
	Operational process	The formal process through which matters are carried out within the organization

**Table 2.** Indices of the structural model for latent variable

Latent variable	R^2^ (%)^1^	CV-Red	CV-Com
Workers’ violations			
Organizational influences	-	0.38	0.38
Unsafe supervision	15.48	0.09	0.67
Preconditions for unsafe acts	23.25	0.24	0.99
Workers’ violations	13.50	0.07	0.42
Workers’ errors			
Organizational influences	-	0.37	0.37
Unsafe supervision	18.10	0.09	0.42
Preconditions for unsafe acts	22.32	0.23	0.99
Workers’ errors	13.22	0.08	0.30
Criteria	2.00, 13.00, and 26.00: small, medium, and large effect, respectively [30]	0<CV-Red [31]

CV-Red, cross-validated redundancy; CV-Com, cross-validated communality.

1 R^2^ is only available for endogenous latent variables.
